# ReactionFlow: an interactive visualization tool for causality analysis in biological pathways

**DOI:** 10.1186/1753-6561-9-S6-S6

**Published:** 2015-08-13

**Authors:** Tuan Nhon Dang, Paul Murray, Jillian Aurisano, Angus Graeme Forbes

**Affiliations:** 1 Department of Computer Science M/C 152, University of Illinois at Chicago, 851 S. Morgan, Room 1120, Chicago 60607-7053, IL, USA

**Keywords:** Pathway visualization, Biological networks, Causality analysis, Topological ordering

## Abstract

**Background:**

Molecular and systems biologists are tasked with the comprehension and analysis of incredibly complex networks of biochemical interactions, called pathways, that occur within a cell. Through interviews with domain experts, we identified four common tasks that require an understanding of the *causality *within pathways, that is, the downstream and upstream relationships between proteins and biochemical reactions, including: visualizing downstream consequences of perturbing a protein; finding the shortest path between two proteins; detecting feedback loops within the pathway; and identifying common downstream elements from two or more proteins.

**Results:**

We introduce *ReactionFlow*, a visual analytics application for pathway analysis that emphasizes the structural and causal relationships amongst proteins, complexes, and biochemical reactions within a given pathway. To support the identified *causality analysis *tasks, user interactions allow an analyst to filter, cluster, and select pathway components across linked views. Animation is used to highlight the flow of activity through a pathway.

**Conclusions:**

We evaluated *ReactionFlow *by providing our application to two domain experts who have significant experience with biomolecular pathways, after which we conducted a series of in-depth interviews focused on each of the four *causality analysis *tasks. Their feedback leads us to believe that our techniques could be useful to researchers who must be able to understand and analyze the complex nature of biological pathways. *ReactionFlow *is available at https://github.com/CreativeCodingLab/ReactionFlow.

## Background

Understanding complicated networks of interactions and chemical components is essential to solving contemporary problems in modern biology, especially in domains such as cancer and systems research [1]. Pathways are essentially subsets of a very large graph; their boundaries are inherently ambiguous, but they are used to limit the scope of analysis. Molecular activation pathways are of critical importance to cancer researchers, who hope to understand - and potentially disrupt - malignant cycles of uncontrolled cellular growth, replication, and mediated cell death [2]. Research in drug development involves determining how proteins affected by a drug in turn affect important cellular pathways, and in this domain the downstream consequences of a particular drug effect are especially important [3]. In a separate domain, stem-cell researchers aim at initiating pathways that will precipitate a desired cellular differentiation into specific cell types [4].

Researchers who work with pathway data are confronted with a number of challenges [5,6]. Pathway files may contain hundreds of proteins and biomolecules (often nested within protein "complexes") that participate in a variety of reactions. In an abstract sense, reactions can be seen as state transitions with multiple inputs and outputs. Participants can act as inputs or outputs to multiple reactions, and the relationships between reactions inherently include feedback loops. Reactions often have an effect on other reactions, inhibiting or promoting their frequency. These molecular activation pathways are inherently dynamic, which limits the utility of any static graph representation [7]. Representing complexity while also enabling researchers to see higher order patterns is a significant challenge [8].

We worked closely over a period of six months with four computational and molecular biologists to identify and prioritize visualization tasks that were important to the biological community. The experts include: a bioinformatician working at a major cancer research lab (designated by the National Cancer Institute as a Comprehensive Cancer Center); a graduate student with a background in both biology and computer science; and two professors in a molecular biology department at a large public university, each running their own research lab. Our in-depth interviews with these experts led us to identify four important tasks that were not currently well-supported in existing visualization tools for pathway analysis. These four tasks each involve reasoning about the causal relationships between proteins and biochemical reactions within a pathway. We refer to these as *causality analysis *tasks, in which an investigation of how upstream and downstream participants of biochemical reactions may lead to new understanding of the nature of the pathway. The four tasks, described in more detail later in the paper, include:

**T1 **Visualizing the *downstream consequences *of perturbing a protein or protein complex;

**T2 **Finding the *shortest path *between two proteins;

**T3 **Detecting *feedback loops *within the pathway;

**T4 **Identifying *common downstream elements *from two or more proteins.

Working with the domain experts led us to design and implement a novel, interactive representation of biological pathways that emphasizes the causality within the pathway, focusing on the biochemical reactions as the "backbone" of the pathway, and highlighting the flow of input and output participants of these reactions through various visual encodings, including animation. Our tool, *ReactionFlow*, visually separates the inputs and outputs of biochemical reactions within a pathway, and emphasizes the relationships between reactions. Visual separation of input and output participants helps reveal highly connected proteins and complexes which may be of particular importance to understanding the nature of a pathway. Perhaps the most useful aspect of *ReactionFlow *involves the representation of *causality *within the pathway, highlighting casual relationships between reactions. We use animation as a means to clearly demonstrate the causal relationships in the pathway.

To augment the visual representation of the pathway with a "reaction-centric" view, we enable a variety of user interactions that make it easier to search through proteins, complexes, or reactions (especially useful in larger pathways), to organize the layout so that reactions are clustered via a topological ordering algorithm or so that edge-crossings are minimized, and to control the animation speed of the flow of activity through the pathway from a selected starting point.

The process of developing *ReactionFlow *was an iterative process that relied on feedback from the four domain experts at various stages of the design and implementation. Many of the ideas that were included were first suggested by one of the domain experts, or emerged organically through these conversations with them. The contributions of this paper are centered around the *ReactionFlow *application and the tasks they enable:

 - We identify four tasks related to understanding causal relationships within a pathway;

 - We introduce a novel visualization to enable causality analysis tasks;

 - We make use of animation to highlight the flow of activity through a pathway;

 - We include mechanisms to search, filter, and order pathways to more effectively present relevant data;

 - We evaluate the effectiveness of our approach through interviews with domain experts.

## Related work

The visualization of biological pathways is challenging due to the complex nature of pathway data, as indicated in the previous section. Given this complexity, publications in molecular biology frequently present biological pathways with human-generated figures. Human creators have the flexibility to arrange visual elements in ways that make representations human readable, and this can allow authors to efficiently encode large volumes of complex information. The human-generated nature of these diagrams allows this complex information to be encoded through clear spatial layouts, organizing the pathway in a meaningful way.

This hand-made approach has been replicated digitally in public databases available online, such as the Kyoto Encyclopedia of Genes and Genomes [9] as well as the Reactome Pathway Database [10], allowing for clear communication and dissemination of established pathways. Several applications exist, such as Entourage [11], that adapt these static figures to be interactive. While these applications preserve the layout and presentation of pathway information, they have several drawbacks. Creating, updating, or modifying figures is a labor-intensive process, and new data cannot be automatically integrated with existing figures. Moreover, these human-generated figures do not easily scale to large, complex pathways. As a pathway increases in size and complexity, human effort to create and to comprehend them also increases considerably. These limitations are major problems in a research community that is continually generating and updating molecular pathway data.

Several tools exist which automatically produce interactive pathway visualizations from structured pathway data, rather than relying on layouts generated by hand. The visualizations produced by these tools mimic the style and visual encoding of human-generated figures in an attempt to to communicate complex network information efficiently. As the layout is computed algorithmically, it is easily updated with new data without requiring extra human effort as networks increase in size. Prominent examples of this approach include Cytoscape [12], ChiBe [13], and VANTED [14]. Both of these tools load pathway data that is represented in a standardized format, such as BioPAX [15]. Users can select from several different layout configurations, and can choose to apply certain visual encodings, such as assigning a color scale to different protein categories.

In addition, ChiBe allows the user to search for proteins of interest, to find relevant pathways, to interactively trace paths between entities, and to identify common regulators or targets of proteins. Finally, experimental data, such as gene expression data or disease association data, can be overlaid on these networks and encoded through color [11]. These features allow researchers to relate pathway visualizations to biological questions more directly than with non-interactive pathway diagrams. Cerebral [16], a Cytoscape plugin, includes visual indications of intercellular context (such as the relative location of the cell wall or nucleus), features that are often included in human-generated diagrams. VANTED enables the user to dynamically edit the network and its layout, and additionally supports the ability to map experimental data to network elements, to perform statistical tests, and to cluster data using machine learning techniques; further analysis can be performed via FluxMap [17], a VANTED plugin.

However, complexity remains a significant challenge for these applications, particularly with respect to the critical task of viewing causality in a pathway. Pathway visualizations show causal direction through directed edges from one event to another, allowing researchers to address questions about what lies downstream of one protein or reaction in a pathway. To see these causal relationships, researchers must trace paths beginning at some starting point and following directed edges through the pathway, but as the number of steps or branches in a pathway increases this task becomes cognitively challenging.

In addition, since these tools mimic the style of pathway diagrams, they do not abstract away details to capture higher-order patterns. For instance, researchers may want to use pathway diagrams to understand the importance of a particular protein in a pathway in order to find suitable targets for drug design. However, critical proteins in a pathway may appear in more than one place in the visualization, perhaps because they are involved in several reactions. To see this, the researcher must either scan visually or use a search tool to find all instances of a protein. Multiple instances of one protein are not immediately evident from the representation, and can be difficult to find through exploration.

Other tools also make use of alternative approaches to representing networks, such as the transition graphs introduced by Pretorius and van Wijk [18], or the interactive bipartite graphs explored by Schulz et al. [19]. Although intended for representing text documents, the design of Jigsaw [20] shares some features with *ReactionFlow*. Jigsaw uses a table-style approach to represent entities of one specific type; related entities that are of different types are connected by links. However, there are basic differences due to specific aspects of pathway datasets. For example, proteins or biomolecules may participate in biochemical reactions directly or they may first bind together in a complex hierarchy before participating. The main visual component of our application can be thought of as a hybrid representation that uses features of network and table layouts.

## Methods

In this section, we describe details about our application, *ReactionFlow*, which provides scalable views of pathway data that reduce complexity while retaining important pathway information relevant for causality analysis tasks. All example biological pathways can be found in the Reactome Pathway Database and are encoded using the BioPAX format. *ReactionFlow *was created over the course of six months via an iterative development cycle in which we made changes or added features based mainly on the feedback from the four domain experts.

### Overview of the *ReactionFlow *application

**Figure 1:**
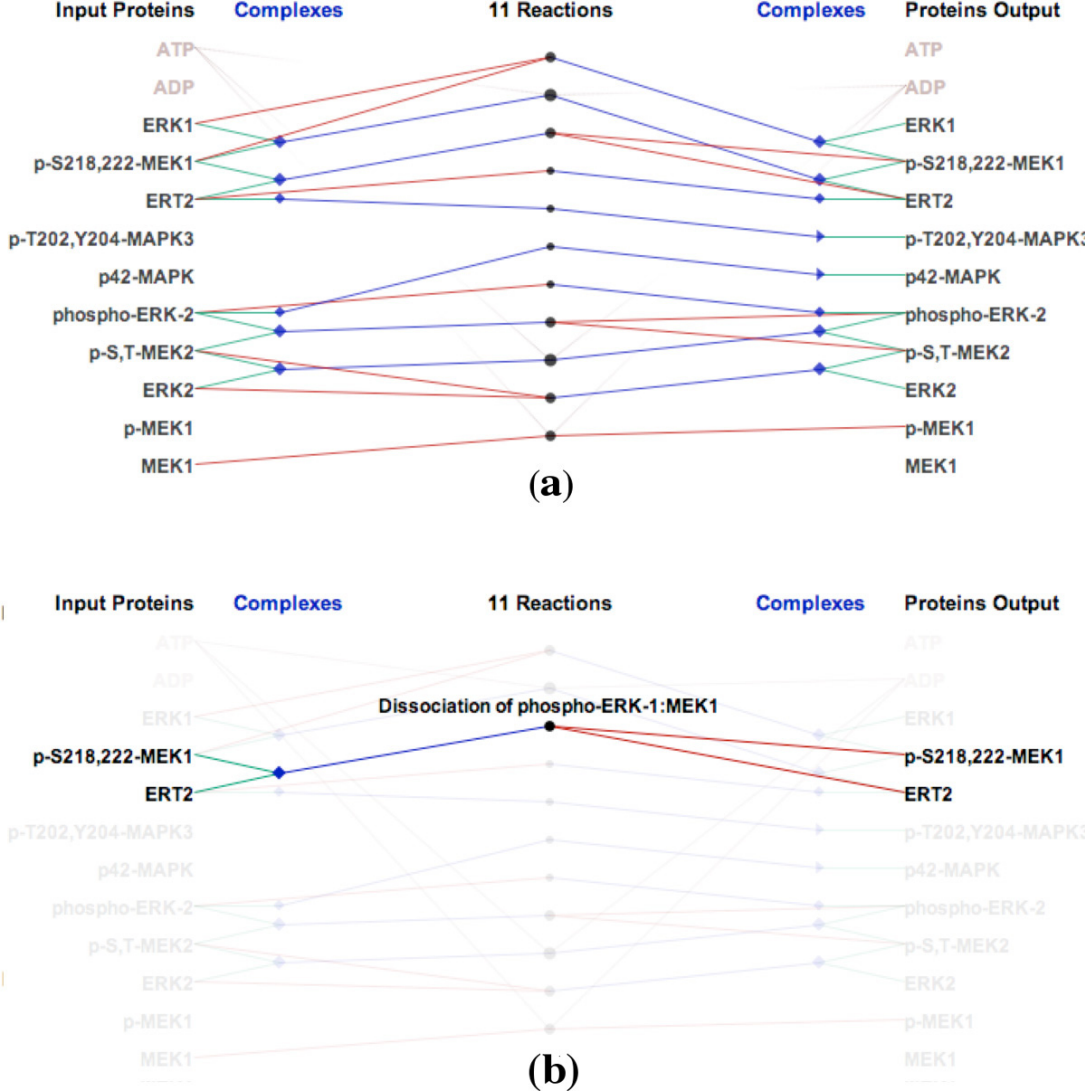
**Visualizing biochemical reactions in the ERK activation pathway: (a) The reaction-centric view of the pathway (b) Brushing a single reaction to highlight its inputs and outputs**.

Figure 1(a) shows an example of *ReactionFlow *displaying several biochemical reactions, in this case using the ERK activation pathway. Within this view - which resembles a parallel coordinates layout - input proteins are listed on the left, and output proteins are listed on the right. Input proteins may directly participate in a reaction (shown with green links) or they may form complexes with other proteins (shown with green links) before participating in a reaction (shown with blue links). Complexes are displayed either as blue diamonds or as blue triangles. Triangles represent complexes that only appear as inputs or outputs, while diamonds represent complexes that act as both inputs and outputs within the pathway. Larger triangles/diamonds indicate complexes containing more proteins. In the pathway shown in Figure 1(a), there are 11 biochemical reactions shown as circles in the center of the view. The circle size is computed based on the number of input and output proteins and complexes of each reaction.

When a reaction is selected via a mouse click, a textual description is displayed, along with the names of all the proteins that participate in this reaction. Figure 1(b) shows an example of brushing the "Dissociation of phospho-ERK-1:MEK1" reaction. In this biochemical reaction, the complex containing p-S218,222-MEK1 and ERT2 is dissociated into two separate proteins. In a similar manner, when we select a protein or complex, *ReactionFlow *highlights all biochemical reactions that it participates in.

**Figure 2:**
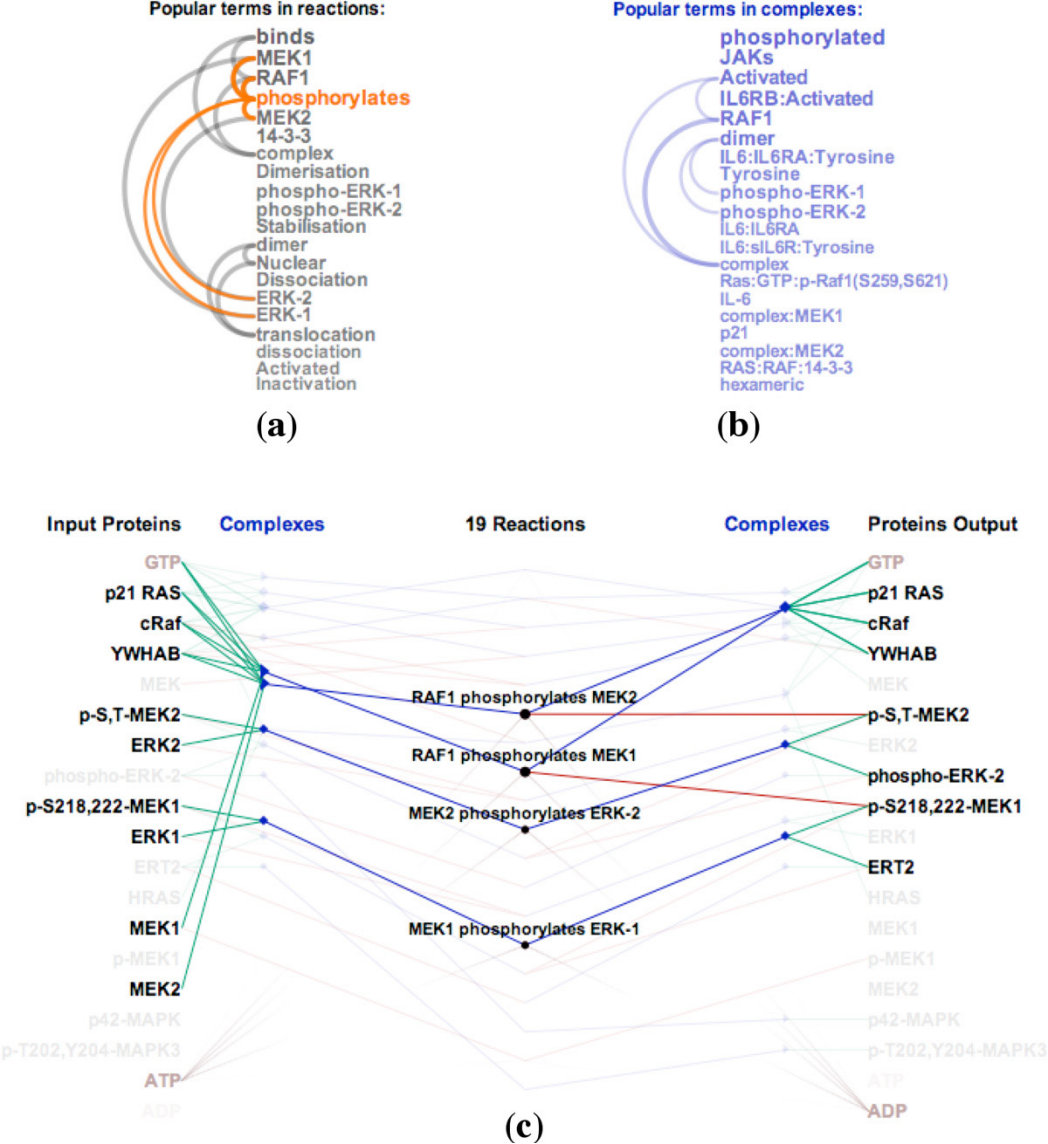
**Visualizing the RAF cascade pathway: (a) Arcs diagram of popular terms in reaction descriptions (b) Arcs diagram of popular terms in complex descriptions (c) Filtering the reactions containing the word "phosphorylates" in their descriptions**.

*ReactionFlow *also includes a search box to filter reactions. As users type a phrase into the search box, reactions and participants that include the typed phrase in their description are highlighted. Typing the word "dissociation", for example, highlights the reactions that dissociate complexes into proteins. A user can also select words from a list of the top 20 most common words that are found in the descriptions of biochemical reactions (shown in Figure 2(a)) or in the descriptions of complexes (shown in Figure 2(b)). The arcs in these lists connect terms that frequently appear together in descriptions of reactions and participants. Hovering over the word "phosphorylates," for example, will highlight reactions that are described containing that term and that therefore involve phosphorylation, as shown in Figure 2(c).

### Introduction to causality analysis in *ReactionFlow*

**Figure 3:**
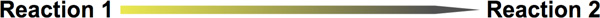
**Gradient colors (yellow to black) are used to indicate the "causality" from reaction 1 to reaction 2**.

Our visualization of biochemical reactions also enables new tasks related to the analysis of causality in biological pathways. In *ReactionFlow*, we have defined a "causal relationship" between two reactions if the *output *participants of one reaction act as the *input *to another reaction. Causality is therefore a directed relationship, and in our visualizations causality is depicted through the use of gradient-filled lines, where direction flows from yellow to black as depicted in Figure 3. In other words, reaction 2 is "downstream" of reaction 1.

**Figure 4:**
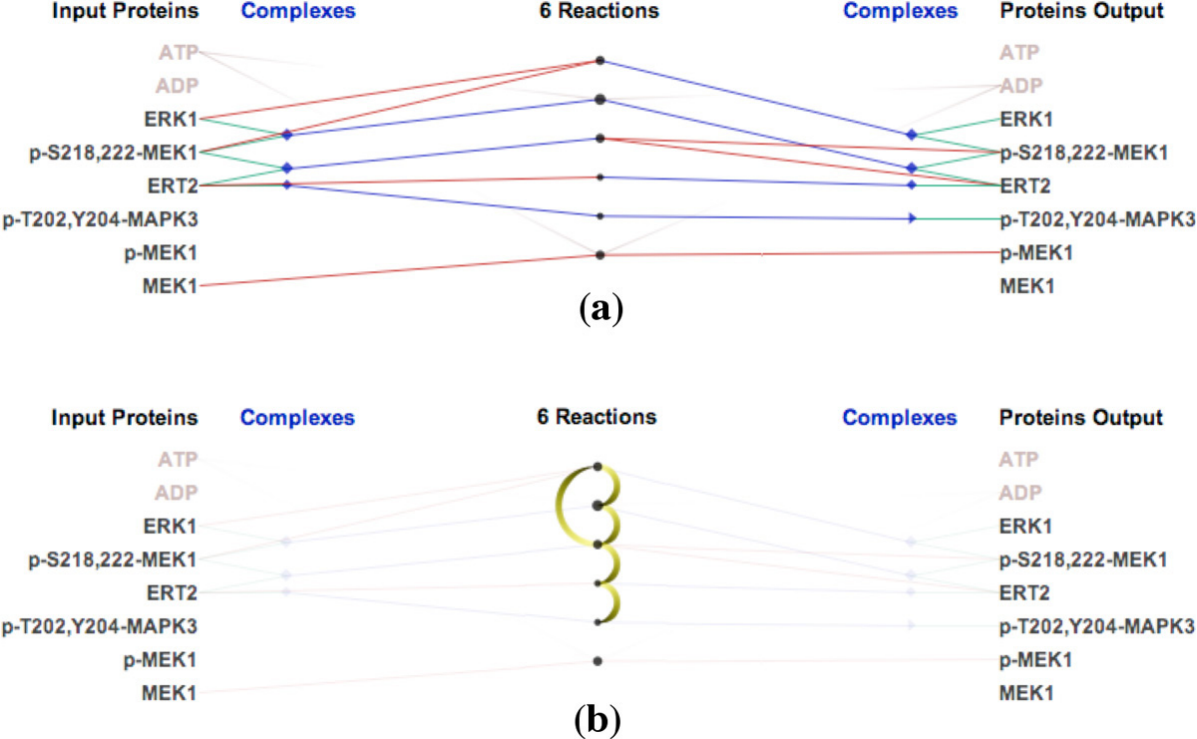
**Causality visualization for the ERK1 activation pathway: (a) The reaction-centric view of the pathway (b) The causal relationships (indicated by the arcs) overlaid on top of the biochemical reactions in the center column**.

**Figure 5:**
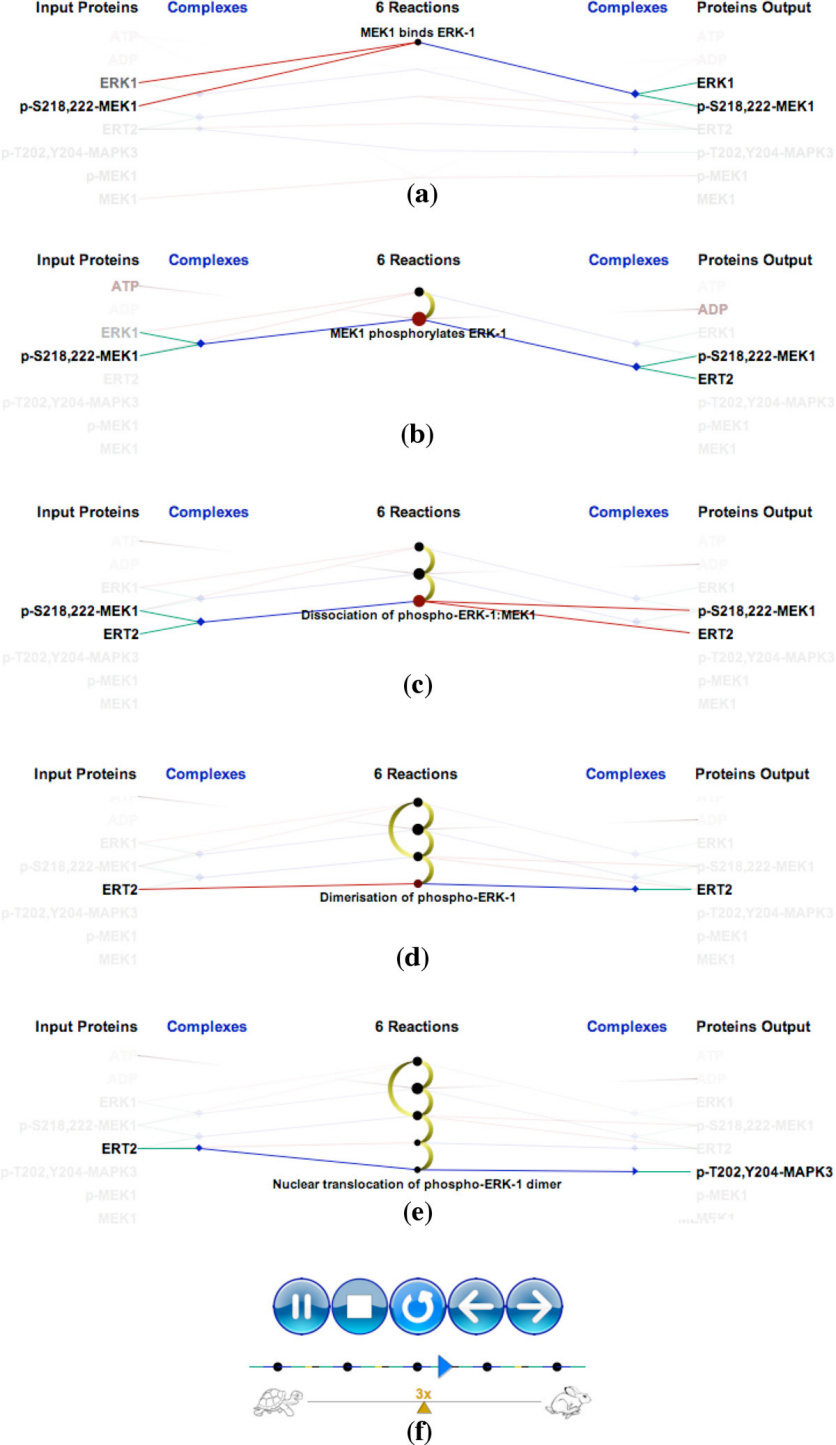
**Animation in the ERK1 activation pathway: (a) The first reaction contains the selected protein ERK1 in the input (b) Second reaction (c) Third reaction (d) Fourth reaction (f) Fifth reaction (e) Animation controllers**.

Figure 4 shows a simple example of the "ERK1 activation" pathway. Figure 4(b) shows 5 causal relationships between 6 reactions in this pathway. "Downstream" reactions are shown with the gradient-filled arcs, which flow from yellow to black along the center column of the visualization. Significantly, we use animation to emphasize the relationship between multiple inputs and outputs in chains of biochemical reactions. Figure 5 shows downstream animation after selecting a reaction in Figure 5(a). The "MEK1 binds ERK-1" reaction produces ERK1 and p-S218,222-MEK1 complex which then serves as the input for the second reaction in Figure 5(b). In the two branches shown in Figure 5(d), only one of them is continually animated since the other one creates a loop back to the first reaction. Figure 5(f) shows a VCR control metaphor which enables the regulation of speed and the mechanism for rewinding the animation. As it is difficult to describe animation in static images, we also invite the reader to refer to the video in our supplementary materials associated with this paper. It should be emphasized that the use of animation is always optional. In cases where animation introduces visual clutter, the user can simply disable this feature. As discussed later in this paper, the use of animation was found to be effective at providing an overview of the causal relations within a pathway.

**Figure 6:**
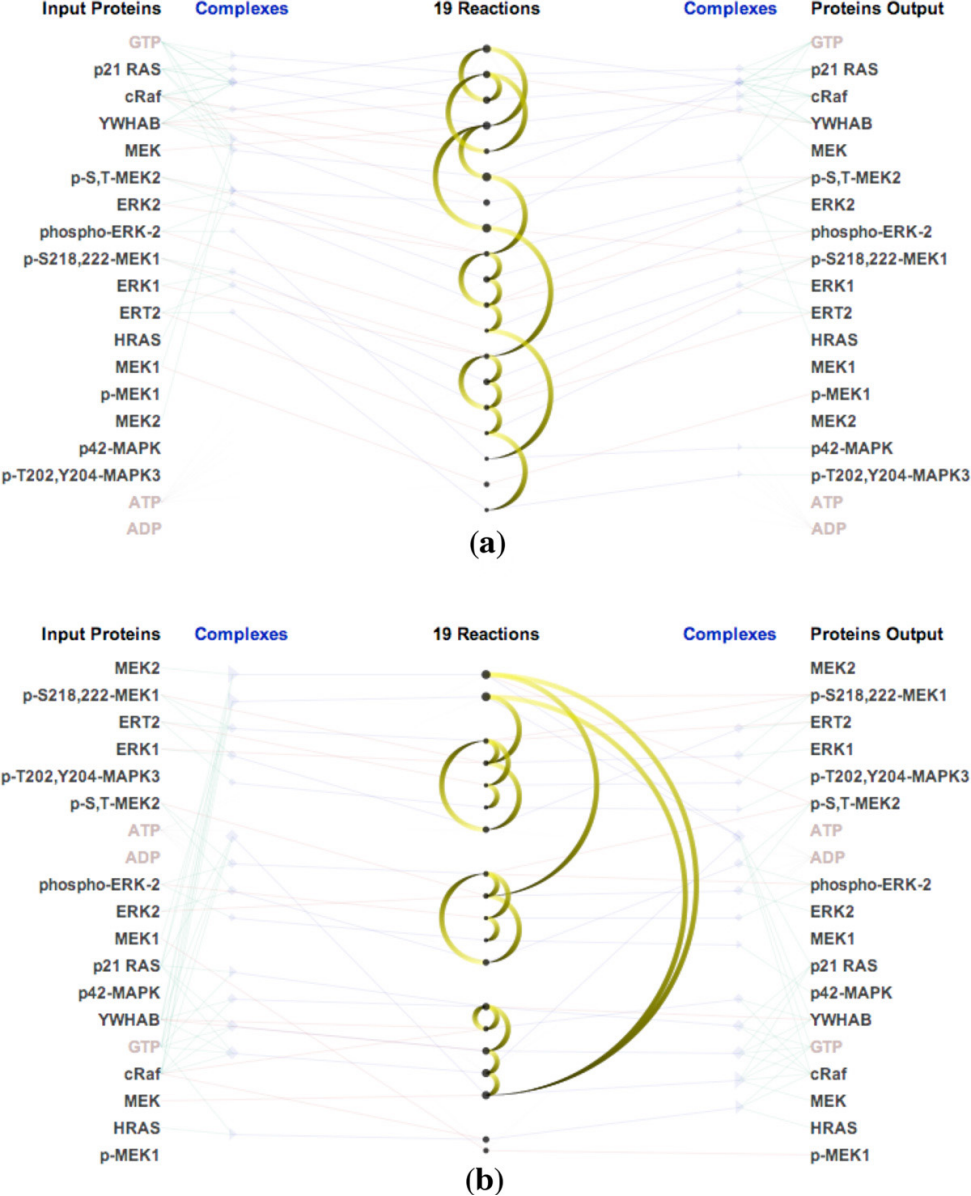
**Ordering reactions in the RAF cascade pathway: (a) Reactions are ordered by their input and output proteins/complexes (b) Topological ordering of causal links**.

Numerous causal arcs are difficult to follow in large pathways. Figure 6(a) shows all causal relationships between 19 biochemical reactions in the "RAF cascade" pathway which itself contains the "ERK1 activation" pathway in the previous example. To make it easier to keep track of visual relationships within the pathway, it is helpful to ensure that downstream reactions appear later in the list. However, this is usually impossible because a reaction can be both downstream and upstream of another reaction, which is very common in pathway data. To address this, we introduce a modification to the topological sorting algorithm (i.e., "toposort") so that it can handle directed cyclic graphs (usually not appropriate when a graph contains directed cycles). Since a pathway may contain cycles, in our modification when there is no node without an input, we instead select the one with maximum outputs (i.e., direct downstream connections) in an attempt to remove as many cycles as possible. This results in a clustering of interrelated reactions, where the clusters tend to be grouped in descending downstream order. Figure 6(b) shows the result of applying our revised topological ordering. This ordering reveals interesting structures within the RAF cascade pathway. The first two reactions ("RAF1 phosphorylates MEK1" and "RAF1 phosphorylates MEK2") belong to the "RAF phosphorylates MEK" sub-pathway. These reactions generate phosphorylated MEK1 and phosphorylated MEK2 which serve as the inputs for two other sub-pathways. The first one is the ERK1 activation pathway which contains the next five reactions in the list. The second one is the ERK2 activation pathway which contains the five next reactions. Notably, ERK1 activation pathway and the ERK2 activation pathway have identical causal structure. Topological ordering of reactions also helps to visually reveal feedback loops. Below we describe key tasks relevant for understanding causality.

### Task 1: Visualizing Downstream Effects

Viewing the downstream components of a biochemical reaction in a pathway helps biologists understand how a drug that perturbs a particular protein might also affect other proteins and complexes that participate in downstream reactions [21].

**Figure 7:**
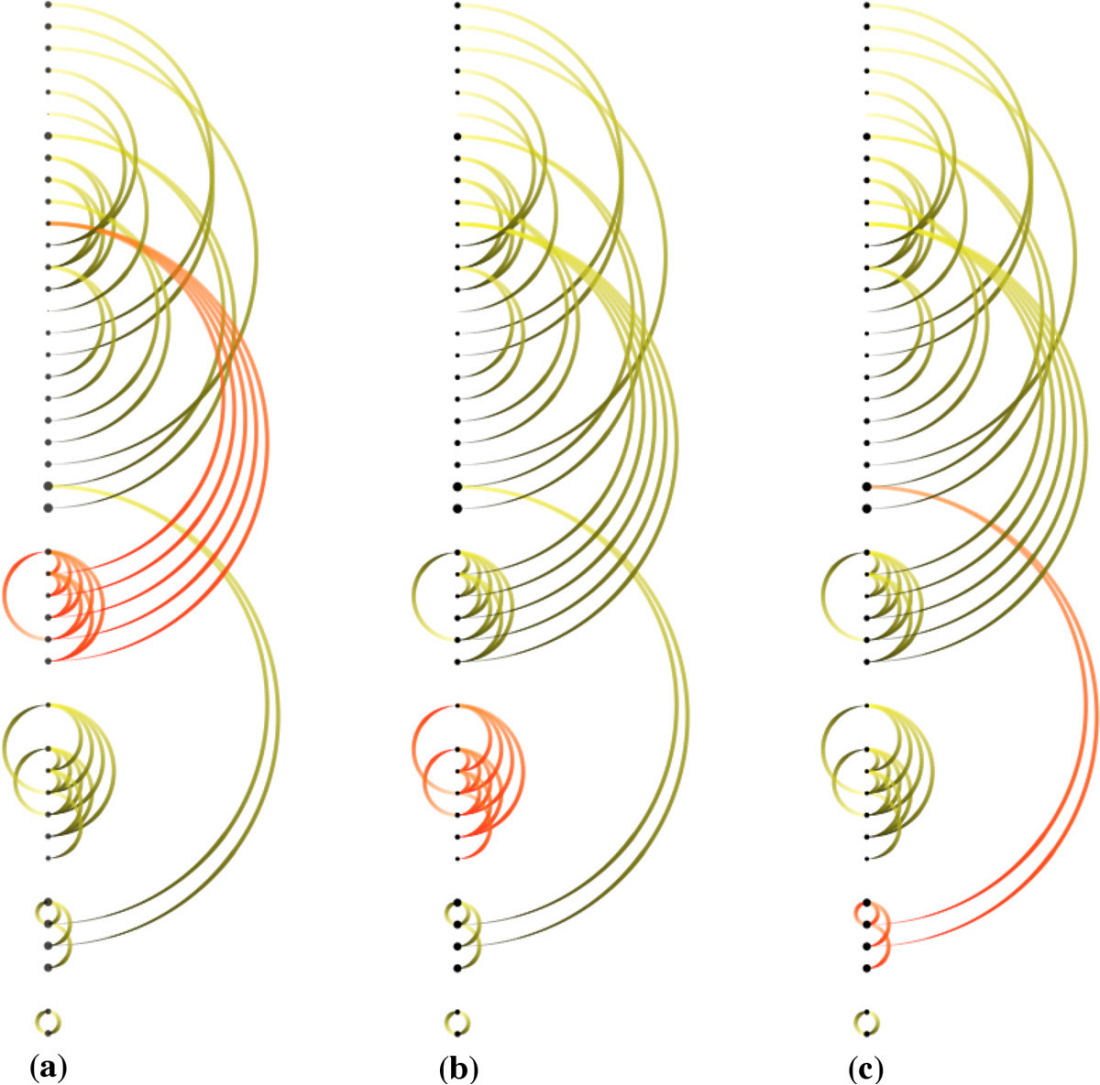
**Downstream consequences if a protein/complex is "knocking out" in the defects in vitamin and cofactor metabolism pathway: (a) GIF protein (b) TCll:Cbl complex (c) MTRR:MTR complex**.

Different proteins play different roles in a pathway. A few proteins control aspects of an entire pathway while other proteins are responsible for a small part (a subset of reactions) in the pathway. For example, the ERK1 protein effects the entire ERK1 pathway while ERT2 is only involved in two reactions. Figure 7 shows examples of how three different proteins are related to downstream reactions and participants in the "vitamin and cofactor metabolism" pathway. In *ReactionFlow*, simply clicking on any of the biochemical components starts an animation that highlights the downstream effects from a given starting point. A user can use the animation tools described above to play, pause, speed up, slow down, or loop through the downstream effects.

### Task 2: Finding the Shortest Path Between Proteins

*ReactionFlow *also provides a tool that highlights the shortest path between two proteins in terms of the number of reactions between an input and an output. When users select an input protein, say, for example, cRaf, *ReactionFlow *displays numbers in front of output proteins that indicate the minimum number of reactions - the number of biochemical "hops" from cRaf - that are needed to generate each output protein from the selected starting point. The color of the links in this view indicate whether steps involve proteins directly (red links) or proteins within a complex (green links). The path from a selected protein to other downstream proteins can also be animated.

**Figure 8:**
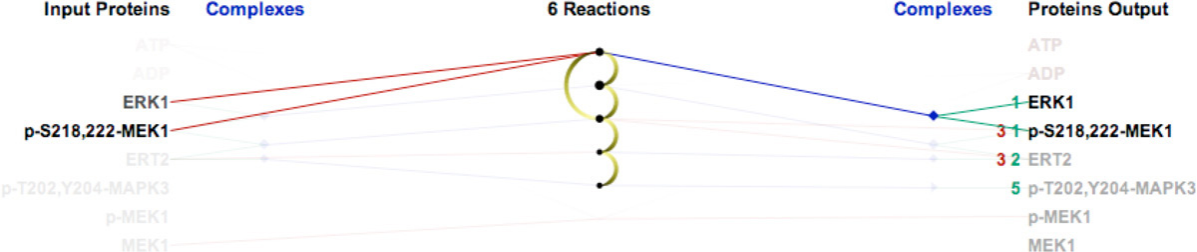
**Shortest path from protein ERK1 to other proteins in the ERK1 activation pathway**.

Figure 8 shows the shortest path from the protein ERK1 to other proteins in the ERK1 activation pathway. The animation begins with the first reaction that is ERK1 is an input into, which ultimately generates the ERK1:p-S218,222-MEK1 complex. We can also reach p-S218,222-MEK1 (as an individual protein) from ERK1 after 3 biochemical reactions. We can also see that p-MEK1 and MEK1 are not downstream participants of the protein ERK1.

### Task 3: Detecting Feedback Loops

**Figure 9:**
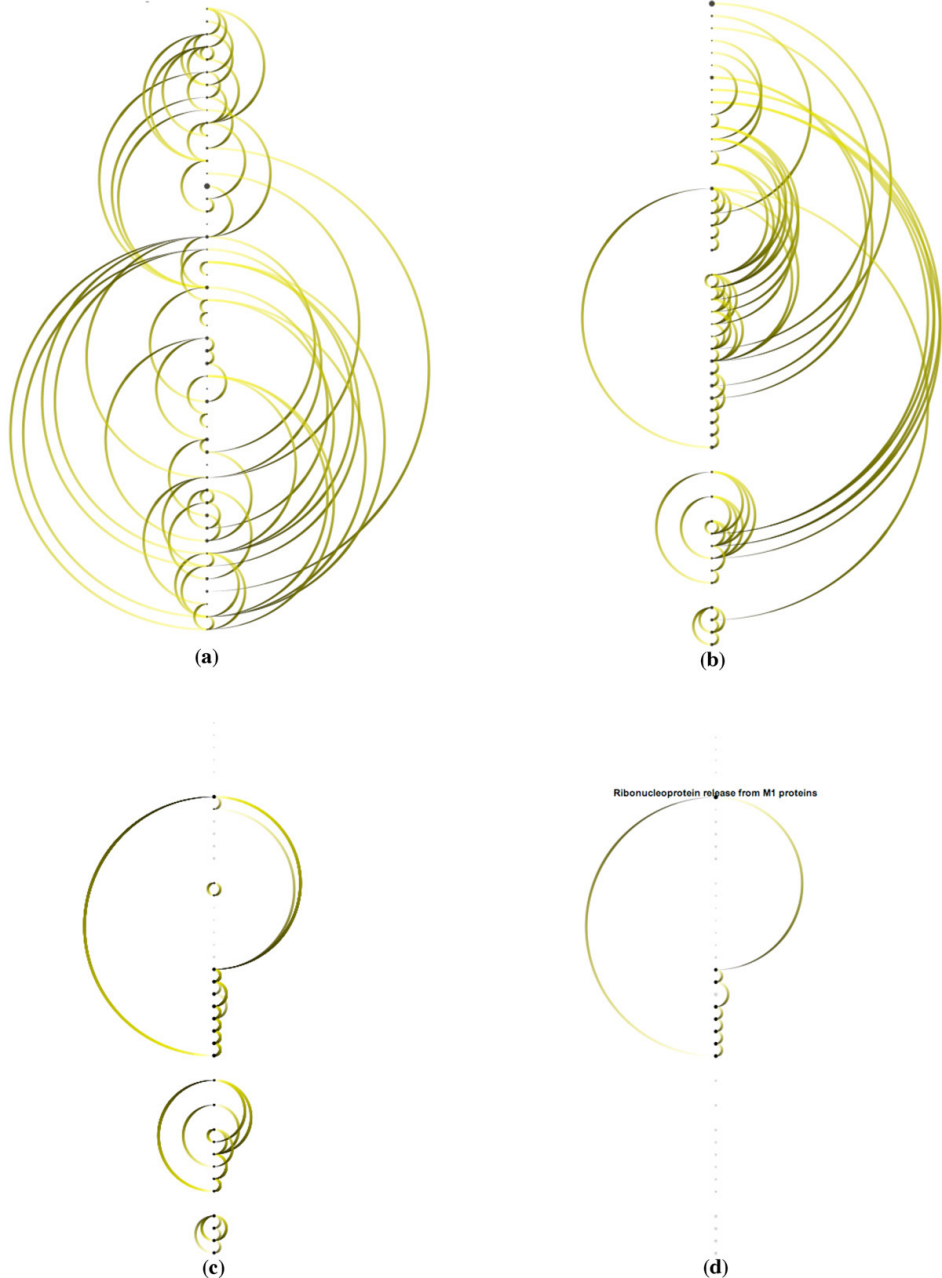
**Feedback loop detection in the influenza infection pathway: (a) All causalities (b) Topological ordering (c) All feedback loops (d) Brushing a feedback loop**.

We can also use *ReactionFlow *to detect feedback loops within a pathway. This makes it easier to identify where cyclical processes are occurring, and thus where perturbations in the network influence both downstream and upstream events. Figure 9 shows feedback loops in the influenza infection pathway (there are 52 biochemical reactions in this pathway). In particular, Figure 9(c) shows all feedback loops. Figure 9(d) shows a selected feedback loop. The benefits of topological ordering can be seen by comparing Figure 9(a) to Figure 9(b), where the topological ordering in Figure 9(b) helps to reveal feedback loops. When the "feedback loop" mode is enabled (via a button on the application), by default all feedback loops are shown, and rolling over any of the reactions in the center column highlights only the feedback loop that reaction is part of (if any).

### Task 4: Finding Common Downstream Elements

An important task for understanding the effects of pathways involves finding common downstream elements that may participate in biochemical reactions. For instance, understanding the functionality of pathways through understanding downstream elements is an active area of cancer research [22-24]. In some cases drugs do not yet exist that target cancerpromoting proteins directly, but but studying pathway data researchers may discover that an existing drug has an indirect influence on cancer-promoting proteins via downstream effects [21]. In other cases, understanding the complex mechanisms behind certain cancers requires an understanding of how multiple activation pathways interact [24].

**Figure 10:**
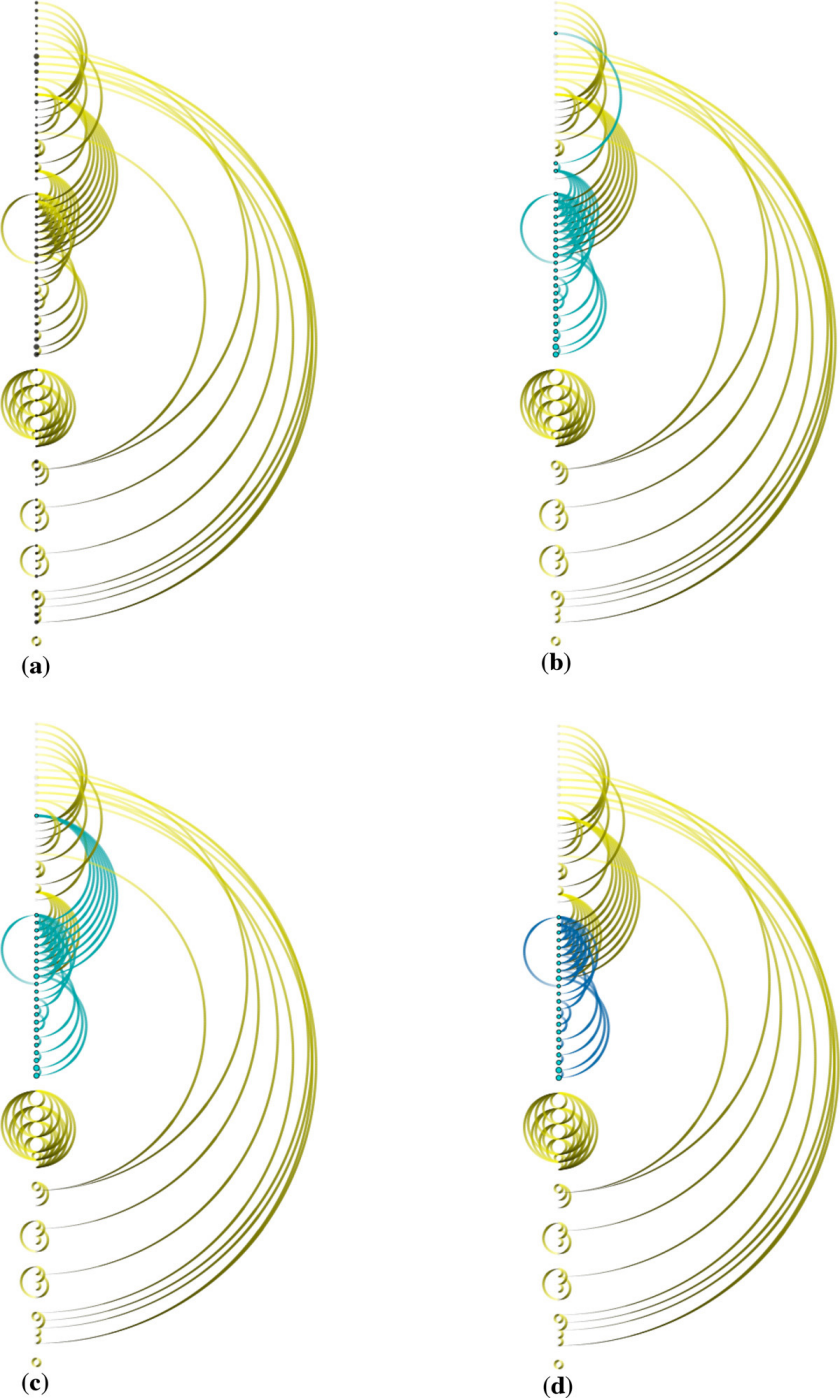
**Example of task 4 on the NGF signaling via TRKA pathway: (a) All causalities (b) Downstream elements of the NTRK1 protein (c) Downstream elements of the Atctive TrkA receptor:Phospho-PLCG1 complex (d) Common downstream elements of the NTRK1 protein and the Atctive TrkA receptor:Phospho-PLCG1 complex**.

*ReactionFlow *provides a "common downstream" function that highlights all reactions that are "downstream" of two or more input participants. Figure 10 shows an example of finding common downstream elements on the "NGF signaling via TRKA" pathway. In particular, Figure 10(b) highlights downstream reactions and their causal relationships of the NTRK1 protein. Figure 10(c) highlights downstream reactions and their causal relationships of the Atctive TrkA receptor:Phospho-PLCG1 complex. Figure 10(d) highlights common downstream elements (in blue) of the NTRK1 protein and the Atctive TrkA receptor:Phospho-PLCG1 complex.

### Reducing visual clutter

Although an aim of *ReactionFlow *is to simplify the visual representation of a pathway, especially to aid in the causality analysis tasks described above, visual clutter can still be an issue, especially in larger pathways containing many proteins and reactions. *ReactionFlow *thus provides various methods to mitigate the visual clutter. For instance, edge crossings can be reduced by reordering reactions and participants, as in Figure 1(b). To order the proteins, we start with a random protein. The next protein is selected if it participates in the same reaction and/or the same complex with the current protein. This selection criteria make sure that proteins in the same complex/reaction are placed next to each other. Consequently, this helps to reduce edge crossings.

**Figure 11:**
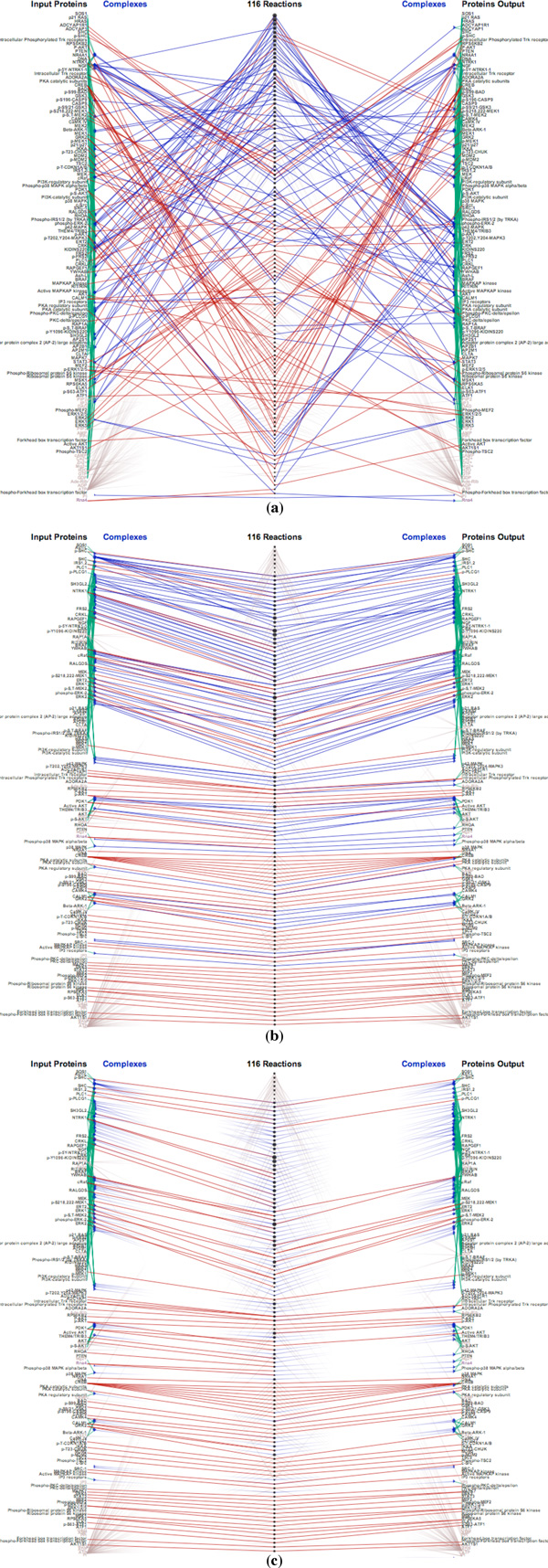
**Visualizing the NGF signaling via TRKA pathway: (a) Random order (b) Order proteins by their reaction/complex participations to minimize edge crossings (c) Fading out reaction links**.

In the next example, we use a larger pathway, *NGF signaling via TRKA from the plasma membrane pathway *containing over a hundred biochemical reactions. Figure 11(a) shows the random order of proteins and reactions. A user can organize proteins in the same complex or reaction so that they remain close together, as in Figure 11(b), significantly reducing edge crossings. Users also have options to "fade out" a specified type (or types) of links in order to make the visualization appear less cluttered, as depicted in Figure 11(c), where the reaction links are tapered and rendered with a low opacity.

**Figure 12:**
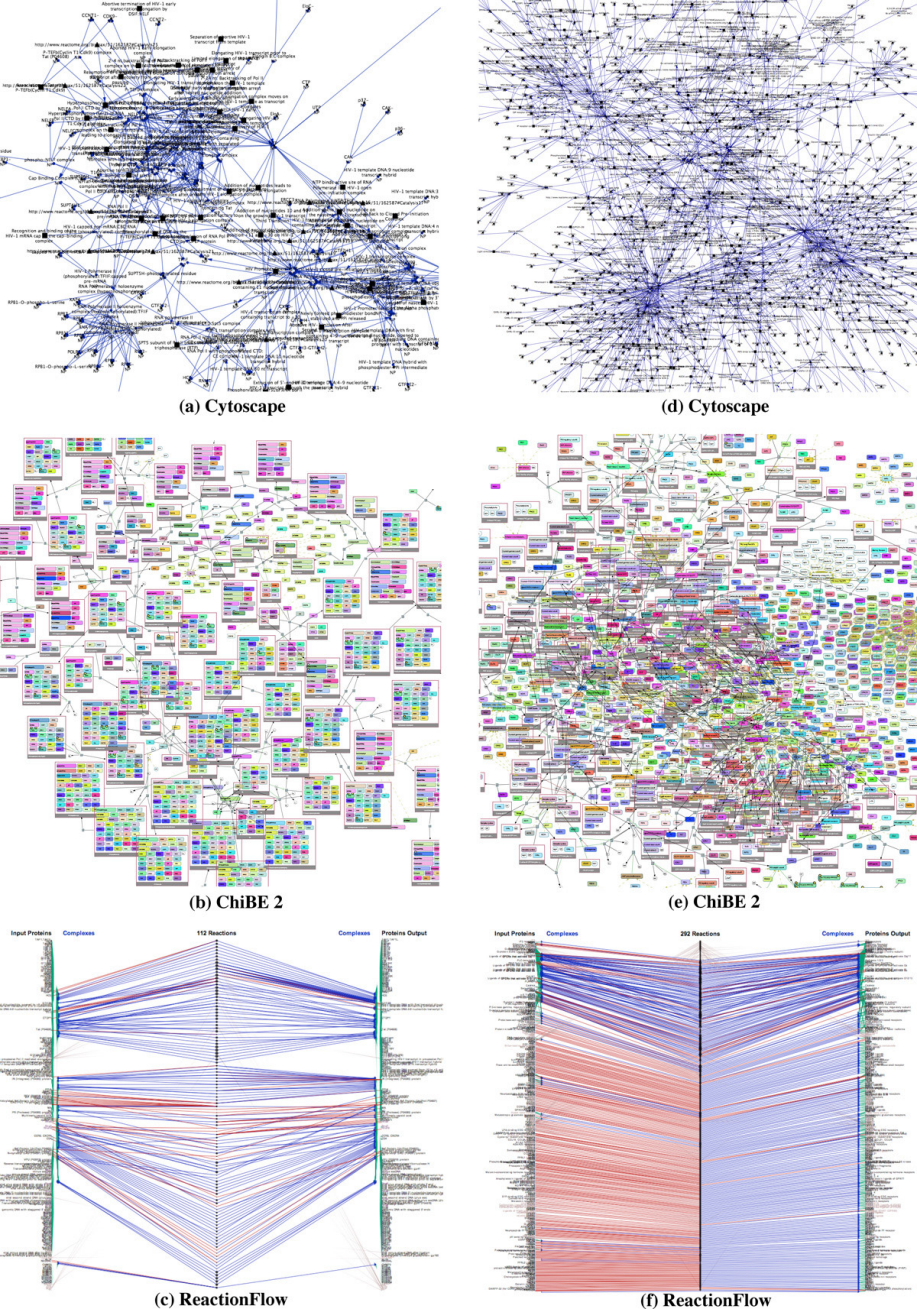
**Visualizing the HIV life cycle (left) and signaling to GPCR pathway (right) by three different tools**.

Regarding the issue of clutter, we compare *ReactionFlow *to other popular tools. Cytoscape features advanced layout algorithms that can minimize edge crossings. Nonetheless, even a medium-sized pathway network, such as HIV life cycle which contains 206 proteins and molecules, 184 complexes, connected via 112 biochemical reactions, demonstrates visual clutter due to link crossing. ChiBE 2 avoids the issue of edge crossing by introducing duplicates of proteins if necessary. However, this makes it more difficult to keep track of reactions involving a selected protein or protein complex (although ChiBE 2 mitigates this somewhat by using different colors to encode proteins). Another problem with node duplication is that for a set of *n *proteins there may be, at least potentially, up to 2*^n ^*complexes, greatly increasing the scale of the network. Figures 12(a) and 12(b) show screenshots of these tools displaying the HIV life cycle. Since *ReactionFlow *pushes proteins and complexes participating into the same reactions together, Figures 12(c) presents two disjoint set of reactions which are not obvious in ChiBE 2. The lower reaction set is associated to the early phrase of HIV life cycle while the upper reaction set is associated to the late phrase of reaction HIV life cycle.

The right panel of Figures 12 shows another example of these tools on a larger input data, the signaling to GPCR pathway. GPCRs stands for G proteinlinked receptors, also known as seven-transmembrane domain receptors. GPCRs are the largest receptor superfamily (with more than 800 G-protein coupled receptor) and also the largest class of drug targets [25]. This pathway contains 413 proteins and molecules, 389 complexes, connected via 292 biochemical reactions. As depicted in the screenshot of *ReactionFlow *shown in Figure 12(c), most inputs of biochemical reactions in this pathway are individual proteins (red links) while most outputs are complexes (blue links). In other words, these are binding reactions of proteins and molecules to form complexes. In fact, there are 157 (out of 292) biochemical reactions belonging to the GPCR ligand binding sub-pathway. Within this sub-pathway, ligands bind to the GPCRs causing conformational changes. These GPCRs can then activate associated G proteins [26]. Since Cytoscape (in Figures 12(d)) and ChiBE 2 (Figures 12(e)) do not separate inputs and outputs, these patterns are not evident even with the same color encodings.

**Figure 13:**
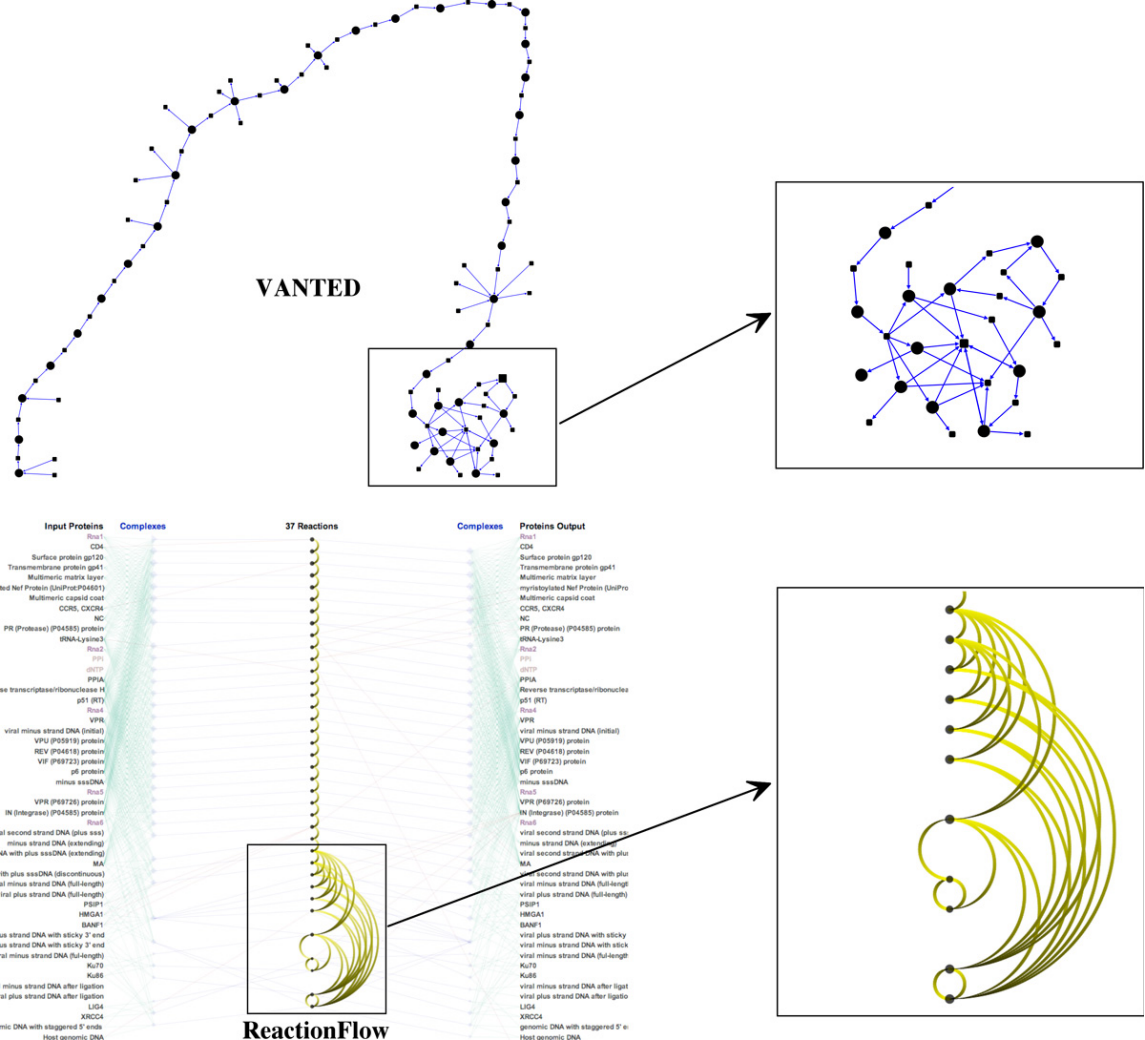
**Visualizing the early phrase of HIV life cycle by VANTED (in the top panel) and ReactionFlow (in the bottom panel)**.

Although we show all edges connecting reactions to input/output proteins and complexes for the comparison to other existing tools, this is not the main interface of our visualization. Instead, a view where only causal arcs connecting biochemical reactions are displayed is the starting point of our pathway analysis. The input/output participants are only displayed on demand for selective biochemical reactions. Therefore, the original tasks are still viable on a system of large size, such as examples in Figures 12. The lower panel of Figure 13 shows the main screen of *Reaction-Flow *when visualizing the early phrase of HIV life cycle. In this figure, we compare our application (in the lower panel) to VANTED (in the top panel). Notice that VANTED displays all biochemical reactions, proteins, and complex participants in the same network. In *ReactionFlow*, we separate input/output proteins and complexes to both sides and reserve the middle section to present causality between reactions in the pathway. This helps *ReactionFlow *to scale more effectively when representing larger networks.

As depicted, both tools successfully reveal the linear nature in the first part of the network (the longest path contains 30 hops). The main difference of two tools is how they display parts of the pathway that contain cycles. A close-up of this sub-network is displayed to the right of each visualization for comparison. Since our revised topological ordering algorithm attempts to push the downstream arcs to the right of the reactions, every arc on the left potentially creates a feedback loop. In this example, we can easily detect the feedback loops associated with these arcs; the same information is not as easy to discern in VANTED without further analysis. Following the recommendation given in a user study conducted by Holten and van Wijk [27], our use of tapered edges also helps to emphasize directionality.

### Implementation and requirements

*ReactionFlow *is implemented in Java and built on top of Processing framework. It is cross-platform, with a minimum requirement of Java 7. *ReactionFlow *uses Paxtools [28] for managing BioPAX data. The application, source code, and sample data are provided via our Github repository, located at https://github.com/CreativeCodingLab/ReactionFlow.

## Results and discussion

We presented *ReactionFlow *to two domain experts, one a molecular biologist and the other a systems biologist, both active researchers highly familiar with particular families of biological pathways that they investigate in their research. We conducted in depth, in person interviews regarding the relevance of our approach to their research tasks, and specifically asked them to carry out each of the four causality analysis tasks. Additionally, we recorded comments about the user interaction and the animation. We also provided a link to the application so that the experts could download the application to their own computers and explore it more at their leisure. We further solicited detailed feedback via email regarding specific functionality related to causality analysis tasks, as well asking for more general feedback about the usability of each of the visualization tools and for suggestions about other features that could be of importance to their research and to other experts in their field.

While we regard this as only first step toward a comprehensive, empirical evaluation of our application and the main visualization techniques it features, we are already encouraged by the positive expert feedback. For instance, both of the experts were excited about how we represented causality. One of them told us: "The causality mapping is very cool. I've not seen anything like it before, and I can see it being very useful." The other noted that the software was surprisingly user-friendly for a prototype application, and appreciated "the causality connecting upstream/downstream reactions and the animation showing each reaction input/output." One of the experts characterized one aspect of his work as "making hypotheses" about the downstream effects of introducing new variables into a pathway, and appreciated that he could quickly mouse over proteins and protein complexes to find the shortest path from an input to output proteins. This expert also provided us with suggestions for additional functionality for *ReactionFlow*, including the ability to load in multiple pathways simultaneously, and also the option to manually select and cluster a group of proteins in order to quickly declutter the view, hiding information less relevant to testing a hypothesis.

One of the experts commented explicitly on the unusual layout, mentioning that he was used to seeing pathways organized as node-link diagrams and saying that "the difficulties in this layout could be with the understanding the flow from left to right, but the animation helps you see how the pathway is organized." After the initial interview, this expert wrote us further comments about the animation: "Animating the flow of biochemical reactions is very useful for understanding causality in the network. Flexible control of that animation is essential, because networks have very different levels of complexity, so some can be viewed quickly, but others must be slowly stepped through." We also asked the experts to comment on the specific tasks. In response to a question for feedback on Task 1, one expert compared our visualization positively to the "gold-standard" of hand-drawn diagrams: "I like hand-drawn diagrams because there is an order... and [*ReactionFlow *] helps capture some of that, which can be lost in some network layouts. Once you figure out what's going on this tool seems more intuitive." When pressed on how an interactive layout could better support the ordering in hand-drawn diagrams, he replied: "You can't completely avoid the complexity - it's inherent. But it's good to get different views that give you some more information from a different perspective."

Regarding Task 2, one expert found out implementation a bit confusing, even after explanations of how it worked. Based on his evaluation, we plan to clarify the visual encoding in a future version of the application. The other expert didn't have trouble understanding the task, but told us that it didn't correspond to his interpretation of what it meant to find the shortest path: "I'd like to be able to click on two proteins and see the shortest path between them." In our current implementation, the user clicks on a single protein, and all downstream components are highlighted, with a number appearing to indicate how many "hops" away it is from the selected protein. The expert thought that provided too much information and was perhaps too similar to Task 1.

Both experts found Task 3 straightforward and easily to understand, and both commented on the importance of identifying feedback loops across multiple pathways, a feature we plan to include in later versions. Regarding Task 4, both experts remarked that it was useful to see which biochemical reactions are "common downstream" components from multiple proteins. One expert told us that one main component of his research was precisely related to this task, which was difficult to visualize in other tools without extensive manual curation: "Sometimes I am interested in understanding how multiple upstream input signals are integrated to drive cellular responses." The other expert also described the usefulness of Task 4: "Families of related proteins are common in human biology. These proteins often share many reactions, but differ in a few key reactions. A view that highlights these commonalities and differences is very useful to parse out these subtleties."

We also asked the experts to point out any other features of *ReactionFlow *which they found to be either effective or ineffective. Both appreciated the grouping of reactions using the topological ordering. One expert noted "it helps to organize the reactions into modules for cleaner layout and visualization." The other also told us: "A key use for pathway representations is to quickly grasp causality. The topological ordering helps a great deal in this by clustering groups of closely related reactions."

Both experts noted that they thought that node-link diagrams, though imperfect in many ways, nonetheless were a more familiar representation for pathways and wondered if that would make it difficult for our tool to be adopted by other biologists. Again, they also both suggested as an obvious next step that we make it possible to load in multiple pathways and moreover then be able to easily remove the elements that were not relevant to them, so as not to overwhelm the visualization with too much information. One expert was initially confused about the use of arcs to indicate relationships in the search terms, since it seemed to clash with the main use of the arcs to illustrate causality between biochemical reactions.

Despite these concerns, both experts were highly positive about the ability to observe and reason about the causality within a pathway. One of the experts also repeatedly expressed his appreciation that our tool emphasized the biochemical reactions within the pathway, since that was a main interest in his own research, and since other visualizations he had used sometimes made it difficult to see only the reactions.

## Conclusions

*ReactionFlow *presents an alternative visual representation of biological pathways, not found in other visualization tools. Our visualization enables a series of novel tasks related to finding patterns within a pathway and to the analysis of causal mechanisms involving proteins and biochemical reactions. Enabling these tasks is potentially very useful to biologists and cancer researchers, as it provides with a means to more deeply understand aspects of biological pathways, such as the downstream connections related to a specific protein complex, or quickly identifying feedback loops.

Our interviews with domain experts show that our visualization techniques, and especially our ability to represent causality in different ways, have the potential to be useful to the larger community of researchers investigating biological pathways. Although we are encouraged by the positive feedback, we have already identified areas for future investigation. For instance, although this paper focuses largely on an alternative representation to a node-link diagram, we believe that it may be possible to present our visualization techniques alongside (instead of in replacement of) a node-link diagram. Although one advantage of our representations is that they are less cluttered than node-link representations, we plan to further investigate the scalability of our system and to explore other ways of interactively compressing or expanding parts of the pathway as needed, something that may prove important for very large pathways containing more than a few dozen reactions. Finally, as suggested by the interviews with domain experts, we plan to explore ways of visualizing multiple pathways simultaneously. A future aim is to enable biologists to understand not just the functionality of a single pathways, but also to help clarify how each pathway functions within a forest of pathways.

## Competing interests

The authors declare that they have no competing interests.

## Authors' contributions

TND, PM, JA, and AGF conceived of the interactive visualization technique and its application to biological pathways. JA provided insight into the datasets used during the development of the technique and TND, PM, JA, and AGF investigated related work. TND and AGF determined the causality analysis tasks through interviews with domain experts. TND designed and implemented all aspects of the prototype visualization tool and provided the comparison to existing tools. TND, PM, JA, and AGF drafted, read, and approved the final manuscript.

## Declarations

This publication is supported by the DARPA *Big Mechanism *Program under ARO contract WF911NF-14-1-0395.

This article has been published as part of *BMC Proceedings *Volume 9 Supplement 6, 2015: Proceedings of the 5th Symposium on Biological Data Visualization: Part 2. The full contents of the supplement are available online at http://www.biomedcentral.com/bmcproc/supplements/9/S6.
